# Skeletal muscle energetics during submaximal contractions are linked to metabolic performance during ambulatory exercise: A pilot study in male adults

**DOI:** 10.14814/phy2.70528

**Published:** 2025-08-26

**Authors:** Christopher M. T. Hayden, Michael A. Busa, Zoe H. Smith, Rajakumar Nagarajan, Luke R. Arieta, Jane A. Kent

**Affiliations:** ^1^ Department of Kinesiology Institute for Applied Life Sciences Amherst Massachusetts USA; ^2^ Center for Human Health and Performance Institute for Applied Life Sciences Amherst Massachusetts USA; ^3^ Department of Medicine University of Massachusetts Chan Medical School Worcester Massachusetts USA; ^4^ Human Magnetic Resonance Center, Institute for Applied Life Sciences University of Massachusetts Amherst Massachusetts USA; ^5^ Present address: University of California Davis Davis California USA; ^6^ Present address: University of Massachusetts Amherst Massachusetts USA

**Keywords:** acidosis, magnetic resonance spectroscopy, mitochondria, muscle oxidative capacity, oxygen consumption, respiratory exchange ratio acetylcarnitine

## Abstract

Skeletal muscle's capacity for oxidative energy production can be measured in vivo by phosphocreatine (PCr) recovery following maximal contractions inside a magnetic resonance scanner. However, muscle energetic characteristics during submaximal contractions of similar intensity as used in free‐living activities may be more relevant to the energetic support of ambulatory tasks during daily life. We measured vastus lateralis muscle *oxidative capacity*, acidification, *submaximal oxidative energy production*, and acetylcarnitine accumulation in response to an incremental contraction protocol (6%–15% maximal torque). We then evaluated the relationships between these metrics and whole‐body metabolic responses to a 30‐min treadmill walk (30MTW) and a peak oxygen consumption (VO_2peak_) test using Spearman rank correlations (*r*_s_, *n* = 7 males, 28 ± 4 years). Muscle oxidative capacity was not related to any metric of whole‐body metabolism, but submaximal PCr recovery was associated with slower VO_2_ off‐kinetics following the 30MTW (*r*_s_ = 0.96, *p* = 0.003). Muscle [acetylcarnitine] was associated with respiratory exchange ratio (RER) during the 30MTW and VO_2peak_ test (*r*_s_ = 0.81, *p* = 0.035 and *r*_s_ = 0.86, *p* = 0.024). Muscle acidification was associated with RER only at VO_2peak_ (and *r*_s_ = ^−^0.89, *p* = 0.012). These preliminary results provide novel connections between muscle and whole‐body energetics and suggest that submaximal muscle energetics may be more relevant to free‐living tasks than oxidative capacity.

## Graphical abstract

Role of muscle energetics in metabolic response to ambulation.

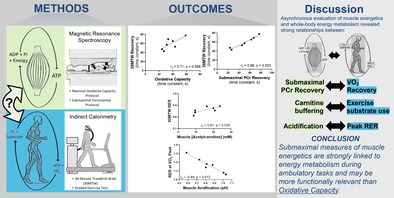

## 1 INTRODUCTION

Mitochondrial dysfunction is a common feature of many health conditions and contributes to a variety of disease‐related pathologies (Pagano et al., 2014). In particular, impaired skeletal muscle oxidative capacity, or the maximal rate of oxidative ATP production by the mitochondria, has been identified in people with diabetes (Phielix et al., 2008), chronic kidney disease (Kestenbaum et al., 2020), and cardiovascular disease (Menon et al., 2021). This impairment is believed to contribute to mobility dysfunction (Choi et al., 2016; Coen et al., 2013; Santanasto et al., 2016; Zane et al., 2017); however, the exact role of oxidative capacity in supporting mobility function has been difficult to define.

Muscle energetics, or ATP production, can be investigated noninvasively and in vivo using ^31^phophorus magnetic resonance spectroscopy (^31^P MRS) to measure changes in intramyocellular pH and high‐energy metabolites such as adenosine triphosphate (ATP) and phosphocreatine (PCr). Because the recovery of PCr following contractions is accomplished exclusively by oxidative phosphorylation, muscle oxidative capacity can be estimated from the rate of PCr recovery following a short bout of maximal contractions that decrease [PCr] by approximately 50% from resting and change in pH no more than ~0.2 (Meyer, 1988; Meyerspeer et al., 2021; Quistorff et al., 1992). Because muscle is responsible for the majority of energy use during exercise, it is likely that a lower oxidative capacity would manifest at the whole‐body level in the form of altered metabolic performance during physical activities.

Metabolic performance during mobility tasks such as walking and running can be measured through respiratory gas exchange via indirect calorimetry. For example, the rate at which VO_2_ decreases following activity (off‐kinetics) is indicative of energetic recovery (Poole & Jones, 2012). Increasing physical fitness leads to faster off‐kinetics (Hagberg et al., 1980; Short & Sedlock, 1997), which should help prevent the progressive accumulation of fatiguing metabolites between free‐living activities and support mobility function–although this has not been empirically tested. Another common metric is the ratio of carbon dioxide production (VCO_2_) to oxygen consumption (VO_2_), termed the respiratory exchange ratio (RER). Under steady‐state conditions, an RER close to 0.7 indicates that fat is the primary substrate being oxidized for energy, whereas an RER near 1.0 indicates that carbohydrate is the primary substrate (Jeukendrup & Wallis, 2005). In general, individuals who are better trained can exercise at greater intensities before transitioning from fat to carbohydrate as a primary substrate for oxidation (Brandou et al., 2005; Dumortier et al., 2003). Measurements of one's ability to adjust substrate use in response to exercise and recover to baseline energy expenditure are strongly associated with physical fitness, mobility function, and exercise capacity (Alexander et al., 2003; Ramos‐Jiménez et al., 2008; Temesi et al., 2017); and reflect both nutrient delivery by the cardiovascular system (e.g., O_2_, glucose, fatty acids) and nutrient use by working muscle (Poole & Jones, 2012). However, the impact of muscle energetic function on substrate use and off‐kinetics during and following whole‐body ambulation has not been determined.

Several groups have evaluated the connection between muscle PCr changes and VO_2_ kinetics during submaximal muscular contractions using simultaneous measurements of ^31^P MRS and VO_2_ (Chilibeck et al., 1998; Rossiter et al., 2000; Rossiter et al., 2002). These studies demonstrated that PCr and VO_2_ on‐ and off‐kinetics mirror each other during single‐leg plantar flexion and knee extension contractions (Chilibeck et al., 1998; Rossiter et al., 2000; Rossiter et al., 2002). However, given the greater muscular involvement and cardiopulmonary demand of locomotion, it is unknown whether such isolated muscle‐group measures are indicative of oxygen kinetics during whole‐body exercise. As such, whether the energetic profile of a single muscle group is indicative of VO_2_ kinetics during ambulatory exercise is not known.

Substrate (i.e., carbohydrate, fat) use at the single muscle level is difficult to evaluate and therefore relate to metabolic performance during walking or running. Using proton (^1^H) MRS, we recently identified a close relationship between intramyocellular acetylcarnitine accumulation and muscle energetics in vivo (Hayden et al., 2024). Because the carnitine system plays an important role in modulating muscle substrate oxidation and metabolic flexibility (Bruls et al., 2019; Miyata, 2013; Muoio et al., 2012; Stephens, 2018; Stephens et al., 2007; Stephens et al., 2013; Wall et al., 2011), muscle carnitine metabolism may also be an important determinant of whole‐body substrate use. While the role of acetylcarnitine has been established at the muscle level (Howlett et al., 1998; Sahlin, 1990), this concept has yet to be fully explored in vivo or translated to the whole‐body level.

Because muscle oxidative capacity has been implicated in mobility dysfunction in aging (Choi et al., 2016; Coen et al., 2013; Santanasto et al., 2016; Zane et al., 2017) and disease (Kestenbaum et al., 2020), we set out to investigate whether muscle oxidative capacity is predictive of substrate use, VO_2_ off‐kinetics, and maximal VO_2_ during walking and running. We also evaluated whether muscle PCr recovery rate following a contraction protocol performed at intensities closer to the knee extensor functional demand during ambulation (Beijersbergen et al., 2013) termed here submaximal PCr recovery–had greater translatability to whole‐body metabolism compared with its oxidative capacity. Lastly, because muscular energetic function is dependent on more characteristics than just oxidative energy production, we also evaluated whether the tendency of muscle to acidify or accumulate acetylcarnitine was related to whole‐body metabolic measures.

To address these questions, vastus lateralis muscle oxidative capacity and energetics were evaluated using MRS in response to a standard oxidative capacity protocol and a submaximal contraction protocol and examined in the context of the whole‐body metabolic responses to a 30‐min treadmill walk (30MTW) and a VO_2peak_ test measured by indirect calorimetry. We hypothesized that: (1) individuals with a lower oxidative capacity and slower PCr recovery following submaximal contractions would have a slower recovery (VO_2_ off‐kinetics) following treadmill walking, a lower VO_2peak_, and a higher RER during both mobility tasks; and (2) greater cytosolic [acetylcarnitine] and lower pH following contractions would be associated with a greater reliance on carbohydrate (i.e., a greater RER) during both treadmill walking and maximal whole‐body exercise, as well as slower off‐kinetics and a lower VO_2peak_. To understand the specific role of muscle in whole‐body metabolism, we completed this pilot study in young healthy adults, thereby minimizing any cardiovascular limitations that are common in aging and disease (Adelnia et al., 2019; Chinnappa et al., 2021; Shimiaie et al., 2015). The results from this initial investigation advance our understanding of the practical implications of *submaximal* muscle energetics and provide insight for future interventions targeting the energetic support of physical activity.

## 2 METHODS

### 2.1 Study overview

Seven healthy males (25–35 years) ranging from sedentary to recreationally active were studied. All participants were non‐smokers and free of any musculoskeletal injury, illness, or medication that could affect neuromuscular or metabolic function. Females were excluded in this pilot study to avoid potential effects of menstrual phase on the metabolic responses to exercise, as we design experiments to properly address this in follow‐up work (Elliott‐Sale et al., 2021). This work was conducted in accordance with the Declaration of Helsinki and was approved by the University of Massachusetts Amherst institutional review board prior to the start of the study. All participants provided written informed consent. Portions of these data have been reported previously (Hayden et al., 2024; Smith et al., 2024).

Participants completed 2 visits to the lab within a 30‐day period, abstaining from alcohol and exercise for 24 h preceding prior, and caffeine the day of each visit. For both visits, participants consumed a standardized lunch meal bar (PROBAR, Park City, UT, 400 kcal, 43 g carbohydrate, 22 g fat, and 11 g protein) at 11:30 am, and experiments (muscle or whole‐body measurements, depending on the visit) commenced at 2:00 pm. The first visit included anthropometric measures, a dual‐energy X‐ray absorptiometry (GE Lunar iDXA, GE Healthcare Lunar, Madison, WI) scan, and a 30‐min treadmill walk (30MTW) during which metabolic gas exchange was measured continuously using indirect calorimetry. After the first visit, participants wore a GT3x accelerometer (ActiGraph, Pensacola, FL) on their dominant hip for 7 days to characterize habitual physical activity levels (Hayden et al., 2024). The second visit included MRS measures at rest and during knee extension contractions, followed by a graded VO_2_ treadmill test to exhaustion (VO_2peak_).

### 2.2 Muscle energetic measures

Muscle energetics were evaluated noninvasively using a surface coil tuned for both ^31^P and ^1^H MRS as previously reported (Hayden et al., 2024). Briefly, ^31^P MRS was used to measure intramyocellular concentrations of inorganic phosphate (Pi), PCr, and ATP before, during, and after contractions. Peaks were fit with Lorentzian lineshapes; one or two peaks were fit to the Pi peak depending on pH, one for PCr, two for the gamma and alpha peaks of ATP, and three for the beta ATP peak (Vanhamme et al., 1997). Because it has the best signal‐to‐noise ratio, [ATP] was determined from the summed area of the gamma peaks. From these measurements, estimates of pH and ATP production by the creatine kinase reaction (ATP_CK_), “nonoxidative” glycolysis, where the end product is lactate (ATP_GLY_), and oxidative phosphorylation (ATP_OX_) were calculated (Hayden et al., 2024). Localized ^1^H MRS provided complementary metabolic information about intracellular acetylcarnitine (Hayden et al., 2024). Participants were positioned supine on the exam bed of a 3‐tesla, 70‐cm, whole‐body MR system (Skyra, Siemens Healthineers, Erlangen, Germany), with their dominant leg secured to a custom‐built leg ergometer (Jaber et al., 2020). A circular, dual‐tuned surface coil assembly (8.5 cm diameter phosphorus and 10.5 cm diameter proton) was secured over the belly of the vastus lateralis muscle and positioned in the isocenter of the scanner. Practice contractions were completed for familiarization with the ergometer. Maximal voluntary isometric torque was calculated from the greatest of three maximal, 3–5 s contractions, each separated by 1 min rest, for establishing the resistance used for the isotonic contractions.

#### 2.2.1 Muscle oxidative capacity protocol

The capacity of the knee extensor muscles to produce ATP oxidatively was evaluated using a protocol consisting of 1 maximal isokinetic contraction (120·s^−1^) every 2 s for 24 s, followed by 10 min of recovery (Bartlett et al., 2024). Participants were instructed to kick as hard and fast as possible through a ~30° range of motion for each contraction and immediately return their leg to the resting position. The recovery of PCr was fit with the following monoexponential equation:

(1) $$\mathrm{PCr}\left(t\right)=Y0+a\cdot \left(1-e^{\left(-\text{kPCr}\cdot t\right)}\right)$$

The variable *Y0* is the average [PCr] over the final 4 s of contractions, *a* is the amplitude of change in PCr, *t* is time, and *k*_PCr_ is the rate constant. All variables were constrained to a minimum value of 0. The time constant (1/*k*_PCr_, s) was used as the measure of oxidative capacity.

### 2.3 Submaximal protocol

After the oxidative capacity protocol, participants completed an 8‐min incremental isotonic contraction protocol, consisting of four 2‐min stages at 6%, 9%, 12%, and 15% of maximal isometric torque, respectively. The goal of this protocol was to evaluate muscle energetic characteristics over a small range of intensities that may be similar to those encountered during daily life. To ensure this protocol remained submaximal, participants were instructed to kick “smoothly and rhythmically” through the range of motion and back in a “consistent manner,” with visual feedback for timing and leg position provided on a monitor.

Intracellular pH was calculated using the chemical shift between PCr and Pi peaks (Taylor et al., 1983):

(2) $$\mathrm{pH}=6.75+\log\left[\left(\text{chemical shift}–3.27\right)/\left(5.69–\text{chemical shift}\right)\right]$$

If changes in pH led to a splitting of Pi into two peaks (Park et al., 1987), both peaks were fit, and an average of the two pH values weighted for the percentage of the Pi pool was used to calculate a single muscle pH (Lanza et al., 2005).

Immediately after the submaximal protocol, participants remained motionless for 10.5 min, during which PCr recovery (min 0–2.5 and ~8–10.5) and post‐contraction [acetylcarnitine] (min 2.5–8) were measured. Submaximal PCr recovery was fit with a biexponential form of Equation 1 (Walter et al., 1999):

(3) $$\mathrm{PCr}\left(t\right)=Y0+\text{a1}\cdot \left(1-e^{\left(-\text{kPCr}1\cdot \text{t1}\right)}\right)+\text{a2}\cdot \left(1-e^{\left(-\text{kPCr}2\cdot \text{t2}\right)}\right)$$

The second term (*+ a2*…) was only included when it yielded a greater *r* ^2^ (*n* = 3); in which cases a single *k*_*PCr*_ was calculated from an average of the two, weighted by amplitude:

(4) $$k_{\mathrm{PCr}}=k_{\text{PCr1}}\cdot \left(\text{a1}/\left(a1+\text{a2}\right)\right)+k_{\text{PCr2}}\cdot \left(\text{a2}/\left(a1+\text{a2}\right)\right)$$

The time constant for PCr recovery (s) was calculated as the inverse of the rate constant for ease of comparison with the whole‐body VO_2_ time constants.

### 2.4 Whole‐body metabolic measures

Whole‐body metabolism was evaluated by gas exchange measures collected on a portable indirect calorimeter (Parvo Medics' TrueOne® 2400, Salt Lake City, UT) throughout the 30 MTW and VO_2peak_ protocols. The cart was calibrated according to manufacturer recommendations immediately preceding each measurement.

### 2.5 Treadmill walk

The 30MTW consisted of 30 min of level walking at 1.3 m·s^−1^ (incline = 0%) with three 1‐min challenge periods (incline = 3% at min 7, 17, and 27). This protocol was used to mimic activity that an individual might encounter during a typical day (Foulis et al., 2017; Hafer et al., 2019). Prior to the walk, participants completed 20 min of quiet sitting followed by 10 min of quiet standing on the treadmill to ensure a steady baseline and quick transition to walking for the on‐kinetics analysis. Immediately following the walk, participants sat for 1 h for the analysis of metabolic recovery off‐kinetics, during which they watched documentary programs and were not allowed to sleep. Gas exchange rates were recorded in 5‐s averages and filtered using a 3‐sample (~15 s) centered moving average. Steady‐state RER was calculated as the mean from min 20–27 of the 30MTW to minimize influence from the challenge periods. Kinetic analysis of oxygen consumption was performed using custom written programs (MATLAB 2020a, The MathWorks Inc., Natick, Massachusetts). The oxygen deficit was fit from the onset of the 30MTW to the start of the first challenge period at 7 min, using the following monoexponential equation:

(5) $${\mathrm{VO}}_{2}\left(t\right)=\left(\text{mean}\;{\mathrm{VO}}_{2}\;\text{final minute of standing}\right)+a\cdot \left(1-e^{\left(-\left(t-d\right)/\mathrm{tc}\right)}\right)$$

where *a* represents the amplitude of change in VO_2_, *t* represents time, *d* represents the time delay between the start of walking and the oxygen response, and *tc* is the time constant (s). All variables were constrained to a minimum value of 0. Oxygen consumption off‐kinetics were fit from the time the participant was seated following the 30MTW to 1 h of recovery, using a similar monoexponential equation.

(6) $${\mathrm{VO}}_{2}\left(t\right)=\left(\text{mean}\;{\mathrm{VO}}_{2}\;\text{final minute of}\;30\mathrm{MTW}\right)–a\cdot \left(1-e^{\left(-\left(t-d\right)/\mathrm{tc}\right)}\right)$$

### 2.6 Peak oxygen consumption

The VO_2peak_ test was performed using a ramped treadmill protocol, as described by Kaminsky & Whaley (1998). Participants completed a self‐paced, 5‐min warm‐up on a treadmill prior to the test, which began with a 0.76 m·s^−1^ flat walk and gradually increased in speed or incline every 20s. Tests progressed until participants reached volitional fatigue. Gas exchange measures were recorded in 5‐s averages, and the modified Borg rating of perceived exertion (RPE; 0–10 scale) was recorded at the end of each minute. During post‐processing, data were filtered using a 6‐sample (~30s) centered moving average to minimize the effect of noise on peak calculation. Peak VO_2_ was then taken as the greatest VO_2_ data point and normalized to lean body mass (mL·kg^−1^ lean mass·min^−1^). The RER at VO_2peak_ was also determined.

### 2.7 Statistics

The hypothesized relationships between measures of muscle energetics and whole‐body energy metabolism were evaluated with non‐parametric Spearman's rank correlation coefficient tests (*r*_*s*_) in GraphPad Prism (version 10.1.2, GraphPad Software, Boston, Massachusetts USA). Precise *p*‐values are reported, and an alpha level for significance of 0.05 was applied.

## 3 RESULTS

**FIGURE 1:**
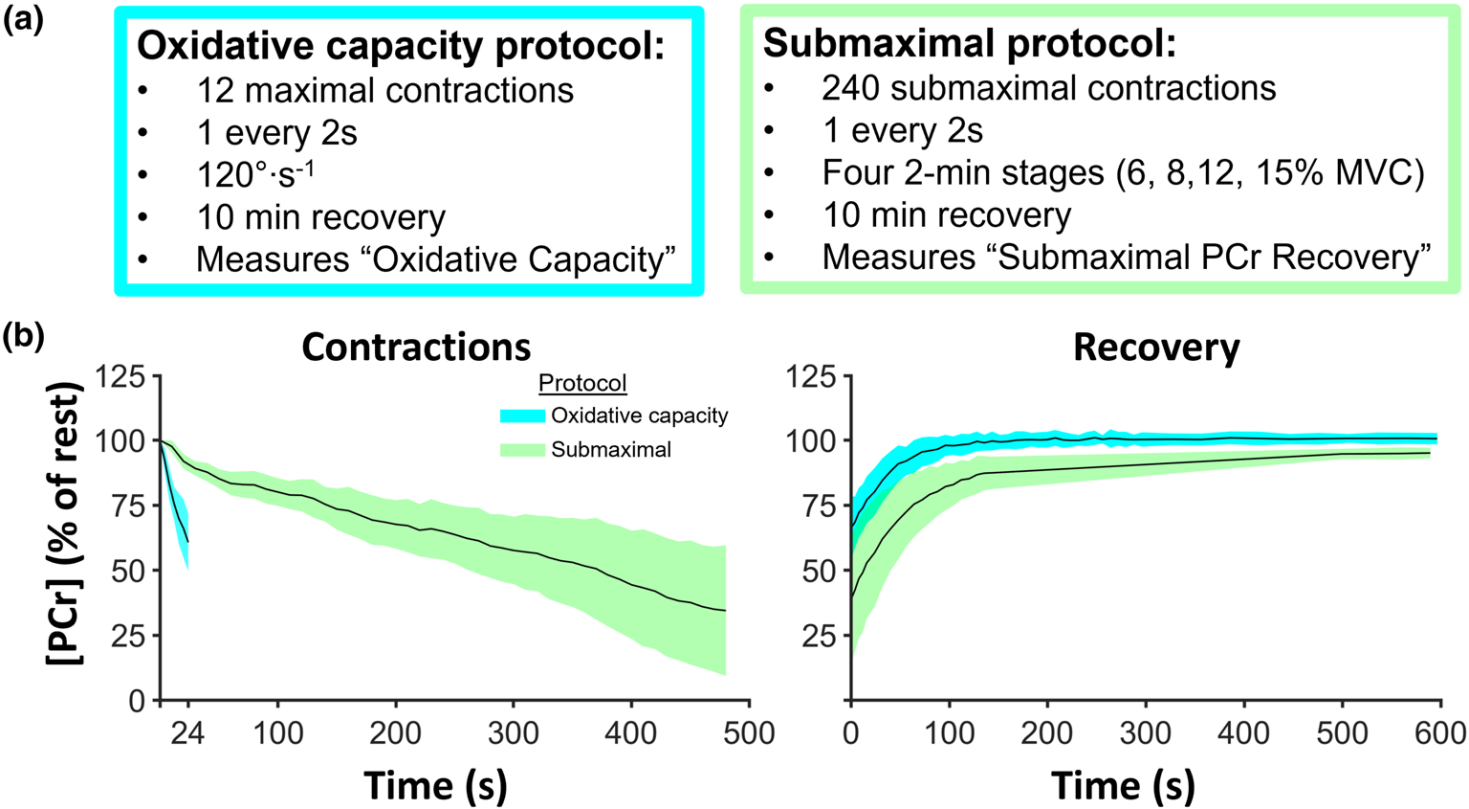
Knee‐extension contraction protocols for muscle energetic measures. (a) Overview of oxidative capacity and submaximal knee‐extension contraction protocols performed on a custom‐built ergometer inside a magnetic resonance scanner, and (b) mean [PCr] normalized to resting values during contractions (left) and recovery (right). Standard deviations are shown in green and blue shading. PCr, phosphocreatine; *n* = 7 healthy young males. Muscle energetics were measured in the vastus lateralis muscle using magnetic resonance spectroscopy at 3T.

**FIGURE 2:**
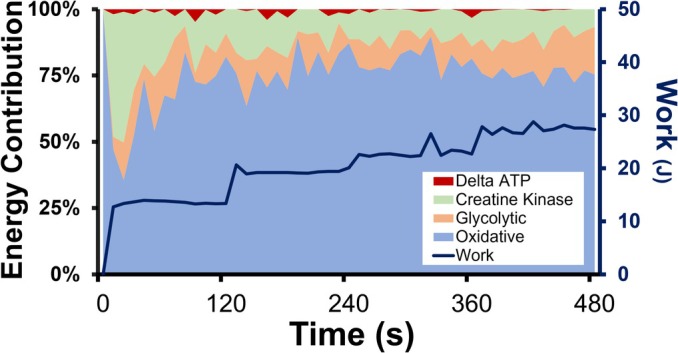
ATP production and work over time during the submaximal knee extension contraction protocol. Vastus lateralis mean energy production from different sources relative to total [ATP] production, and work performed during the 8‐miN submaximal contraction protocol. Standard deviations are omitted for clarity. *n* = 7 healthy young males. Muscle energetics were measured using magnetic resonance spectroscopy at 3T.

**FIGURE 3:**
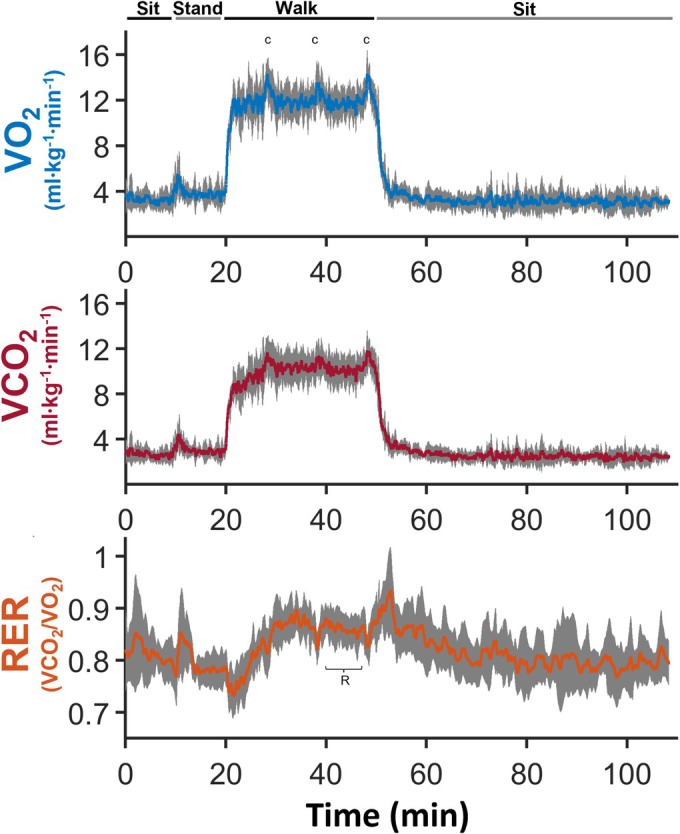
Whole‐body metabolic variables over time during the 30MTW. Mean VO_2_, VCO_2_, and RER over time in response to 30 min of treadmill walking at 1.3 m·s^−1^. Standard deviations are shown in gray. The protocol started with ~20 min of sitting (only the first 10 min are shown in order to temporally align the data), followed by 10 min of quiet standing, 30 min of walking, and 60 min of seated recovery. VO_2_, volume of oxygen consumed; VCO_2_, volume of carbon dioxide produced; RER, respiratory exchange ratio; 30MTW, 30‐min treadmill walk; *n* = 7 healthy young males. Oxygen consumption was measured via metabolic cart.

Anthropometric and summary data from the muscle MRS and whole‐body gas exchange measures are shown in Table 1. Mean PCr responses for the oxidative capacity and submaximal protocols are shown in Figure 1. During the oxidative capacity protocol, PCr breakdown was the primary source of ATP, while during the submaximal protocol (Figure 2), the contribution of each pathway to ATP generation varied with intensity and time. Mean VO_2_ in response to the 30MTW increased ~three‐fold, from 3.7 ± 0.8 mL·kg^−1^·min^−1^ while standing to 12.0 ± 1.3 mL·kg^−1^·min^−1^ during min 7–30 of walking, which was 29 ± 5% of VO_2peak_. The mean time‐course responses of VO_2_, VCO_2_, and RER during the 30MTW are shown in Figure 3. Mean VO_2peak_ normalized to total body weight was 42.6 ± 6.1 mL/kg/min. Test duration ranged from 11 to 16 min, and all tests were terminated upon volitional fatigue. No whole‐body outcome was significantly correlated with any other whole‐body outcome (e.g., VO2_peak_ was not correlated with off‐kinetics; all *p* values ≥0.088).

**TABLE 1:** Summary data.

	Mean ± SD	Range
Participant characteristics (*n* = 7)		
Age (years)	28 ± 4	25–35
Height (cm)	180 ± 10	164–189
Body mass (kg)	90 ± 16	65–105
Body fat (%)	22.9 ± 5.9	13.8–34.7
Lean mass (kg)	65.9 ± 12.2	47.4–84.8
Daily step count	5224 ± 2367	3491–10,001
30‐min treadmill walk		
Steady‐state VO_2_ relative to VO_2peak_ (%)	28.6 ± 5.1	23–36
VO_2_ off‐kinetics time constant (s)	59.0 ± 12.5	42.9–77.7
Mean RER, min 20–27	0.86 ± 0.03	0.81–0.90
VO_2_ peak protocol		
Absolute VO_2peak_ (L·min^−1^)	3.84 ± 0.83	2.49–4.90
Relative VO_2peak_ (mL·kg^−1^ lean mass·min^−1^)	58.3 ± 7.6	51.6–72.8
RER at peak VO_2_	1.17 ± 0.09	1.06–1.31
24‐s muscle oxidative capacity protocol		
Oxidative capacity time constant (s)	36.9 ± 11.8	24–58
8‐min submaximal contraction protocol		
PCr recovery time constant (s)	50.2 ± 20.2	23.3–76.9
Post‐contraction [Acetylcarnitine] (mM)	15.5 ± 6.2	8.7–23.6

Abbreviations: PCr, phosphocreatine; RER, respiratory exchange ratio; VO_2_, volume of oxygen consumed.

### 3.1 Associations between single‐muscle energetics and whole‐body metabolism

#### 3.1.1 VO_2_ off‐kinetics

**FIGURE 4:**
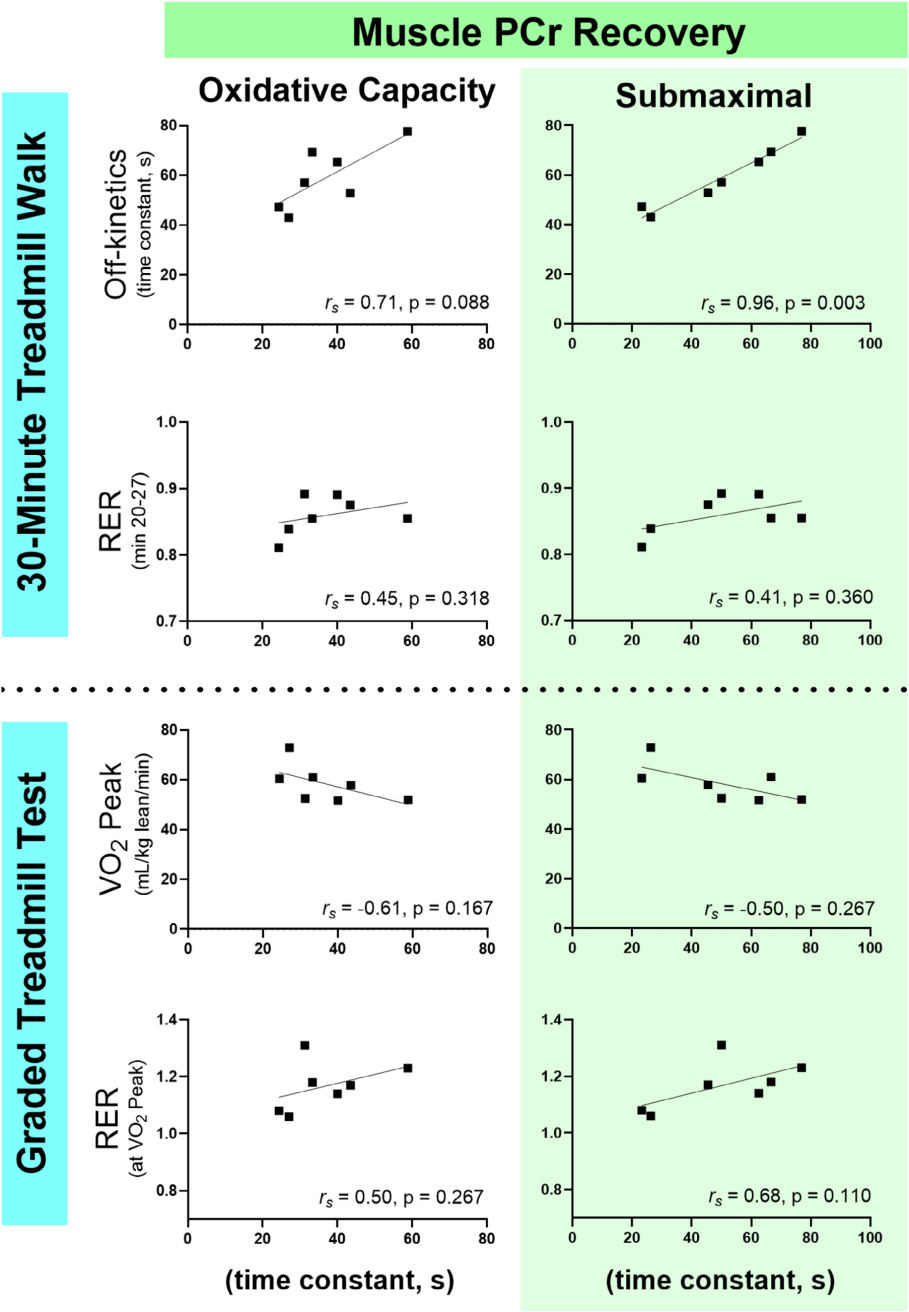
Associations between whole‐body metabolic performance and PCr recovery time constants from oxidative capacity and submaximal contraction protocols. The relationships between whole‐body VO_2_ off‐kinetics and RER from the 30MTW and VO_2peak_ tests with the time constant for muscle PCr recovery following the oxidative capacity and submaximal knee extension contraction protocols. RER, respiratory exchange ratio; 30MTW, 30‐min treadmill walk; PCr, phosphocreatine; VO_2_, volume of oxygen consumed; VO_2peak_, greatest VO2 consumption during the graded exercise test; r_s_, Spearman's rho: *n* = 7. Oxygen consumption was measured via metabolic cart. Muscle energetics were measured in the vastus lateralis muscle using magnetic resonance spectroscopy.

**FIGURE 5:**
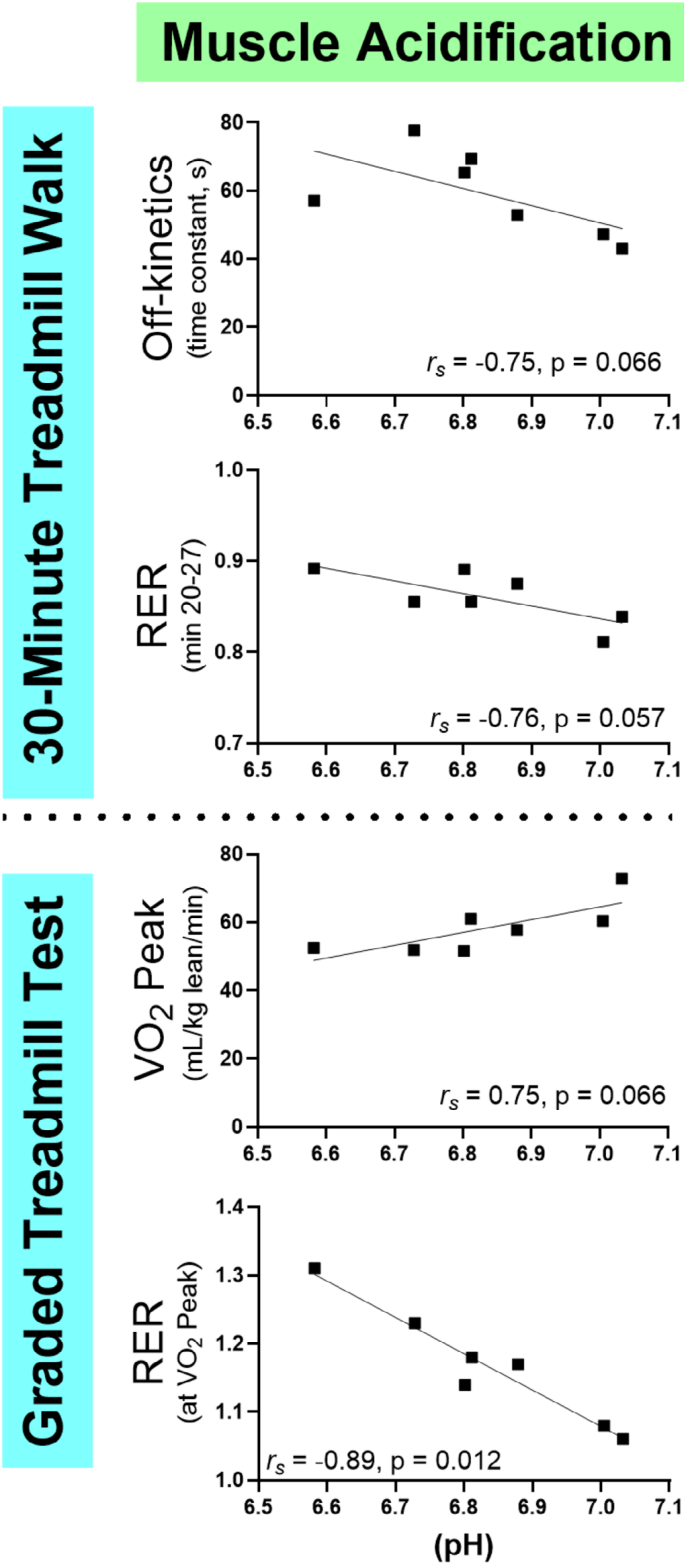
Associations between whole‐body metabolic performance and muscle acidification from the submaximal contraction protocol. The relationships between whole‐body VO_2_ off‐kinetics and RER from the 30MTW and VO2_peak_ tests with muscle pH at the end of the submaximal knee extension contraction protocol. 30MTW, 30‐min treadmill walk; RER, respiratory exchange ratio; VO_2_, volume of oxygen consumed; VO_2peak_, greatest VO_2_ consumption during the graded exercise test; r_s_, Spearman's rho: *n* = 7. Oxygen consumption was measured via metabolic cart. VO_2peak_ was measured on a treadmill. Muscle energetics were measured in the vastus lateralis muscle using magnetic resonance spectroscopy.

**FIGURE 6:**
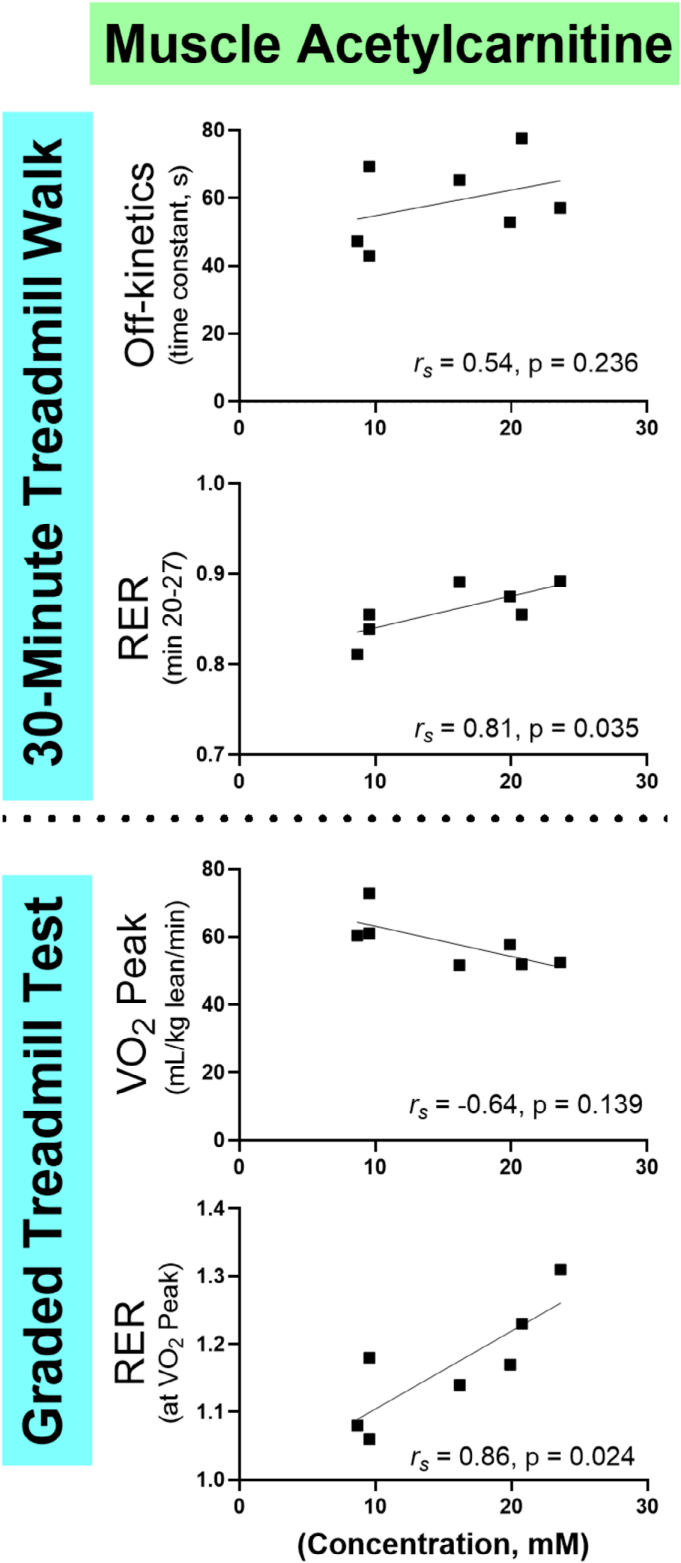
Associations between whole‐body metabolic performance and muscle acetylcarnitine accumulation following knee extension contractions. The relationships between whole‐body VO_2_ off‐kinetics and RER from the 30MTW and VO2_peak_ tests and muscle [acetylcarnitine] following the submaximal knee extension contraction protocol. 30MTW, 30‐min treadmill walk; RER, respiratory exchange ratio; VO_2_, volume of oxygen consumed; VO_2peak_, greatest VO_2_ consumption during the graded exercise test; r_s_, Spearman's rho: *n* = 7. Oxygen consumption was measured via metabolic cart. VO_2peak_ was measured on a treadmill. Muscle energetics were measured in the vastus lateralis muscle using magnetic resonance spectroscopy.

The time constant for VO_2_ return to baseline following the 30MTW was associated with the time constant for PCr recovery following the submaximal contraction protocol (Figure 4). Muscle oxidative capacity, acetylcarnitine accumulation, and acidification were not associated with off‐kinetics (Figures 4, 5, 6). These results suggest that oxidative PCr resynthesis during submaximal contractions may be more relevant to metabolic recovery from whole‐body physical activity than muscle oxidative capacity.

#### 3.1.2 VO_2peak_

None of the muscle energetic outcomes were significantly related to VO_2peak_ (Figures 4, 5, 6). Only the relationship between VO_2peak_ and muscle acidification approached significance.

#### 3.1.3 Respiratory exchange ratio

The steady‐state RER during the 30MTW was associated with intramyocellular [acetylcarnitine] following the submaximal contraction protocol (Figure 6), but not with muscle acidification (Figure 5), illustrating that muscle carnitine metabolism may influence whole‐body substrate use during ambulation. The RER at VO_2peak_ was associated with both intracellular [acetylcarnitine] and acidosis in response to the submaximal protocol (Figures 5, 6). These relationships bolster the concept that whole‐body energy substrate use during high‐intensity exercise is established in part by inherent muscle energetic characteristics that reflect ATP production (acidosis) and selection of substrate for oxidative phosphorylation ([acetylcarnitine]).

## 4 DISCUSSION

The purpose of this preliminary study was to investigate whether the inherent energetic characteristics of a major locomotor muscle are indicative of whole‐body metabolism during ambulatory exercise. We did so by evaluating whether knee extensor muscular energetics were associated with the metabolic responses to submaximal and maximal treadmill exercise in the same group of participants. We found that individuals who recovered more slowly after walking (VO_2_ off‐kinetics) had slower PCr recovery following a submaximal muscle contraction protocol, while muscle oxidative capacity was not significantly correlated with off‐kinetics. Individuals with a greater RER (more use of carbohydrate) during the 30MTW had greater intramyocellular [acetylcarnitine] following the submaximal knee extension protocol. Additionally, RER at VO2_peak_ was positively correlated with both intramyocellular [acetylcarnitine] and muscle acidification following the submaximal knee extension protocol. Together, the results from this preliminary study identify novel intrinsic aspects of *submaximal* muscle energetics that are associated with important metrics of whole‐body energy metabolism during both submaximal and maximal locomotor tasks.

### 4.1 VO_2_ off‐kinetics, VO_2peak_ , and substrate use

In general, the “excess” O_2_ consumed post‐exercise is believed to be due to the replenishment of oxygen stores; oxidative regeneration of PCr; residual elevations in ventilation, heart rate, body temperature, and catecholamine levels; conversion of lactate to glucose; glycogen synthesis; the re‐establishment of ionic equilibrium; and, potentially, an increased protein turnover (Gaesser & Brooks, 1984; Gore & Withers, 1990). Off‐kinetic times in this study ranged from ~43 to 78 s, suggesting participants would be fully recovered in ~2–4 min. While VO_2_ off‐kinetics can reflect metabolic performance across various intensities, VO_2peak_ is a measure of the body's maximal capacity for oxidative metabolism and is a common metric of general fitness (Kaminsky et al., 2022). Our participants' average VO_2peak_ fell between the reference values for 20–29 and 30–39 year old males, which matches their average age of 28 (Kaminsky et al., 2022). Substrate use at a given intensity is variable across the population but is relatively invariant in an individual (Jeukendrup & Wallis, 2005). Mean RER increased from ~0.7 to just below 0.9 over the first ~10 min of the 30MTW before attaining a plateau at ~0.85 during min 20–27 (Figure 3), suggesting that the metabolic signal that controls substrate use might take time to reach steady state. All participants reached an RER >1.05 during the VO_2peak_ test, which indicates that bicarbonate buffering of hydrogen ions was contributing to CO_2_ production (Beaver et al., 1986a, 1986b). These hydrogen ions are produced by muscle acidification, and an RER above 1.1 is a standard indicator of maximal effort during whole‐body exercise.

### 4.2 Muscle oxidative capacity and submaximal PCr recovery

We found a strong positive relationship between VO_2_ off‐kinetics and the time constant for PCr recovery following the submaximal contraction protocol (Figure 4), suggesting that faster muscle recovery following 8 min of contractions (i.e., shorter PCr recovery time constants) facilitates more rapid recovery of VO_2_ after whole‐body activity (i.e., shorter off‐kinetic time constants). These results extend previous work showing that simultaneous measures of VO_2_ and PCr time constants mirror each other during isolated limb exercise (Chilibeck et al., 1998; Rossiter et al., 2002) by using asynchronous measures to detect novel links between energetic characteristics in a single locomotor muscle and whole‐body metabolism during human locomotion. Somewhat surprisingly, VO_2_ off‐kinetics were not significantly related to muscle oxidative capacity (Figure 4), although this may have been due in part to our small sample size. Typically, oxidative capacity protocols are designed to evaluate maximal oxidative ATP production in an unfatigued, minimally perturbed state (i.e., PCr depletion of ~50% and pH decline <0.2; (Meyerspeer et al., 2021)). In contrast to the muscle oxidative capacity protocol, which yielded an average PCr recovery time constant of ~37 s, the average PCr recovery time constant from the submaximal contraction protocol was ~50 s (Table 1). This slowing of oxidative ATP production following dynamic contractions has been described previously following this (Smith et al., 2024) and other protocols (Bartlett et al., 2020; Walter et al., 1997), which has been attributed to changes in pH affecting the creatine kinase reaction (Kemp et al., 1994; Lawson & Veech, 1979; Meyer et al., 1991; Sahlin et al., 1979).

The significant relationship between VO_2_ off‐kinetics and PCr recovery following the submaximal protocol, but not the oxidative capacity protocol, suggests that the energetic state of the muscle following the submaximal protocol more closely resembles muscle during daily activity, such as walking, than does the oxidative capacity protocol. Thus, while oxidative capacity reflects peak mitochondrial ATP production, it appears that this may be less pertinent to everyday activities than submaximal muscle energetics, at least in our sample of healthy young males.

Neither muscle oxidative capacity nor submaximal PCr recovery were associated with VO_2peak_ (Figure 4). This is in contrast to several previous studies that found associations between VO_2_ peak normalized to total mass and muscle oxidative capacity estimated by in vitro respiration (*r* ^2^ = 0.81–0.89) (Van Der Zwaard et al., 2016), near‐infrared spectroscopy (*r* ^2^ = 0.57) (Lagerwaard et al., 2019), and ^31^P‐MRS (*r* ^2^ = 0.54) (Adelnia et al., 2019). It has been suggested that VO_2peak_ normalized to total body mass may have greater implications for exercise performance, while VO_2_ normalized to lean mass (more metabolically active tissue) is more relevant when discussing physiological capacity (Goran et al., 2000). This is logical, as an increase in body fat would increase the workload of a graded treadmill test, but not the oxidative capacity of the muscle. Oxidative capacity and submaximal PCr recovery were also not significantly related to RER during the 30MTW or the VO_2peak_ test, suggesting that substrate use is not solely dependent on the muscle's ability to produce ATP oxidatively.

### 4.3 Muscle acidosis

During the 8‐min submaximal contraction protocol, muscle acidified modestly to an average pH of 6.81, consistent with the submaximal nature of the contractions. Acidification (H^+^ accumulation) occurs in skeletal muscle when the rate of glycolysis exceeds the rate at which the mitochondria take up and oxidize its product, pyruvate. Hydrogen ions that are not buffered within the muscle efflux into the blood and are buffered in part by sodium bicarbonate, leading to CO_2_ production. As such, RER (VCO_2_/VO_2_) not only reflects carbohydrate oxidation but also H^+^ buffering and, thus, non‐oxidative glycolysis. We found that muscle acidification during isolated contractions was strongly related to a greater RER at VO_2_ peak (Figure 5), suggesting that the proclivity of muscles to acidify even under submaximal conditions may partly explain RER patterns during maximal whole‐body exercise. In contrast, during low‐intensity walking exercise, RER more likely reflects oxidative carbohydrate use, which may explain the apparent lack of significant association between muscle acidification and RER during the 30MTW (Figure 5). Although muscle acidification did not significantly correlate with VO_2_ off‐kinetics, 30MTW RER, or VO_2peak_, the p value for these relationships was ≤0.066 in these cases (Figure 5), suggesting that a larger sample size may reveal an important role for intramuscular pH in whole‐body metabolic performance.

### 4.4 Acetylcarnitine

A limitation of ^31^P MRS for investigating muscle energetics is that the separate contributions of fat and carbohydrate oxidation to ATP production cannot be determined. The carnitine system has long been suggested to play a role in substrate selection (Stephens et al., 2007), and in a recent paper using portions of these data, we found that post‐contraction [acetylcarnitine] was highly correlated with glycolytic ATP production (Hayden et al., 2024). It is thus possible that acetylcarnitine production may serve as a marker for substrate oxidation during in vivo MRS studies as previously suggested in ex vivo work (Howlett et al., 1998; Sahlin, 1990), with greater [acetylcarnitine] indicating a greater reliance on carbohydrates. This concept is supported here indirectly by the observation that individuals with greater post [acetylcarnitine] relied more on carbohydrates for fuel during the 30MTW and at VO_2peak_ (Figure 3a,c). These data provide impetus to explore muscle acetylcarnitine production as a proxy for muscle substrate oxidation in vivo, which could be verified through concurrent H^+^ MRS and gas exchange measures. Acetylcarnitine accumulation was not associated with off‐kinetics or VO_2peak_, which are indicators of general fitness (Figure 6). However, [acetylcarnitine] in resting muscle has been shown to vary with fitness levels in some but not all previous studies (Klepochova et al., 2017; Klepochová et al., 2018; Lindeboom et al., 2014), suggesting that further research about acetylcarnitine metabolism during exercise, particularly in combination with additional techniques that query other aspects of energetics and metabolism, is warranted.

### 4.5 Additional considerations

We note several limitations to this study, including the small sample size, cross‐sectional and correlational design, and narrow participant demographics. However, the novelty of these results gives rise to a number of questions that may be pursued with a larger prospective study designed to clarify the role of submaximal muscle energetics in human locomotion, and the implications of these factors on mobility and health in a variety of populations. Further, the small and relatively homogeneous sample here highlights the sensitivity of these muscle metrics to small differences in whole‐body metabolism that would likely only grow with the inclusion of populations with greater variations in health and fitness.

### 4.6 Conclusions

We provide novel evidence that the inherent *submaximal* energetic characteristics of a single muscle, rather than its oxidative *capacity*, are linked to whole‐body energy metabolism during locomotion. We also present preliminary evidence for relationships between whole‐body substrate use and both muscle acidification and acetylcarnitine accumulation, identifying muscle energetic characteristics that may be important determinants of whole‐body substrate selection. Finally, the consistency of the relationships between muscle acidosis and each of the whole‐body metabolic metrics supports the inclusion of muscle pH measures in future studies addressing the links between muscle energetics and free‐living energy metabolism. Collectively, these results support the idea that whole‐body metabolism during exercise is dependent on additional muscular factors outside the maximal ability to generate oxidative energy.

## AUTHOR CONTRIBUTIONS

Christopher M.T. Hayden, Michael A. Busa, and Jane A. Kent conceived and designed research; Christopher M.T. Hayden, and Zoe H. Smith, performed experiments; Christopher M.T. Hayden and Rajakumar Nagarajan analyzed data; Christopher M.T. Hayden, Michael A. Busa, Zoe H. Smith, Luke R. Arieta, and Jane A. Kent interpreted results of experiments; Christopher M.T. Hayden prepared figures; Christopher M.T. Hayden drafted manuscript; Christopher M.T. Hayden, Michael A. Busa, Zoe H. Smith, Rajakumar Nagarajan, Luke R. Arieta, and Jane A. Kent edited and revised manuscript. All authors approved final version of manuscript.

## FUNDING INFORMATION

Portions of this project were funded by the UMass Amherst Institute for Applied Life Sciences Voucher Program.

## CONFLICT OF INTEREST STATEMENT

None of the authors have any conflicts of interest to disclose.

## ETHICS STATEMENT

All experimental protocols were reviewed and approved by the University of Massachusetts Amherst Institutional Review Board and all experiments conducted in accordance with the Declaration of Helsinki.

## Data Availability

The data that support the findings of this study are available from the corresponding author upon reasonable request.
